# A Cross-Sectional Survey of Population-Wide Wait Times for Patients Seeking Medical vs. Cosmetic Dermatologic Care

**DOI:** 10.1371/journal.pone.0162767

**Published:** 2016-09-15

**Authors:** Geeta Yadav, Hanna R. Goldberg, Morgan D. Barense, Chaim M. Bell

**Affiliations:** 1 Faculty of Medicine, University of Toronto, Toronto, Canada; 2 Division of Dermatology, Women’s College Hospital, Toronto, Canada; 3 Department of Psychology, University of Toronto, Toronto, Canada; 4 Institute for Clinical Evaluative Sciences in Ontario, Toronto, Canada; 5 Departments of Medicine and Health Policy Management and Evaluation, University of Toronto, Toronto, Canada; 6 Department of Medicine, Mount Sinai Hospital, Toronto, Canada; San Gallicano Dermatologic Institute, ITALY

## Abstract

**Background:**

Though previous work has examined some aspects of the dermatology workforce shortage and access to dermatologic care, little research has addressed the effect of rising interest in cosmetic procedures on access to medical dermatologic care. Our objective was to determine the wait times for Urgent and Non-Urgent medical dermatologic care and Cosmetic dermatology services at a population level and to examine whether wait times for medical care are affected by offering cosmetic services.

**Methods:**

A population-wide survey of dermatology practices using simulated calls asking for the earliest appointment for a Non-Urgent, Urgent and Cosmetic service.

**Results:**

Response rates were greater than 89% for all types of care. Wait times across all types of care were significantly different from each other (all P < 0.05). Cosmetic care was associated with the shortest wait times (3.0 weeks; Interquartile Range (IQR) = 0.4–3.4), followed by Urgent care (9.0 weeks; IQR = 2.1–12.9), then Non-Urgent Care (12.7 weeks; IQR = 4.4–16.4). Wait times for practices offering only Urgent care were not different from practices offering both Urgent and Cosmetic care (10.3 vs. 7.0 weeks).

**Interpretation:**

Longer wait times and greater variation for Urgent and Non-Urgent dermatologic care and shorter wait times and less variation for Cosmetic care. Wait times were significantly longer in regions with lower dermatologist density. Provision of Cosmetic services did not increase wait times for Urgent care. These findings suggest an overall dermatology workforce shortage and a need for a more streamlined referral system for dermatologic care.

## Introduction

Wait times for medical care have become a “hot-button” issue for many Western countries as populations age and demand for health care services increases [1, 2]. Much research has also been done comparing wait times between the United States and Canada, with most studies suggesting wait times are generally shorter in the United States [3]. In 2013, Canada ranked last among the 11 OECD countries in terms of wait times for an appointment with a family doctor and wait times to be seen in an emergency department [4]. Recent studies have also noted long wait times for dermatology appointments, an issue which will become even more pressing given that the number of dermatology visits in the US alone is projected to increase by 16% between 2013 and 2025 [5]. Others observe similar trends with demand outstripping supply due to retired practitioners and increased need [6]. This trend will likely be exacerbated in rural areas [7].

Increasing demand for medical dermatologic care has occurred at the same time as a rise in cosmetic dermatology services provided by many of those same practitioners [8, 9]. Though previous work has examined some aspects of the dermatology workforce shortage and access to dermatologic care, little research has addressed the effect of rising interest in cosmetic procedures on access to medical dermatologic care [10, 11]. In the US, one study found that dermatologists offered faster appointments to patients calling about cosmetic procedures than they did for those about melanoma [12, 13]. In the Canadian health system, physician’s offices, and not patients themselves, make referrals to specialists (i.e. patients do not require a referral for cosmetic services). All essential medical care is covered by a universal insurance plan without deductibles, whereas cosmetic services are instead paid for out of pocket. The universal health care system provides an ideal context to assess the effect of cosmetic services on medical care because reimbursement rates are standardized and access is not limited. Thus, we aimed to compare the wait times for Urgent, Non-urgent, and Cosmetic dermatology services in order to examine whether the wait times for medical dermatologic care are affected by the offering of cosmetic services within a practice and to determine potential influencing factors.

## Methods

Our study population consisted of all dermatologists who had a primary practice located in Ontario and were registered with the College of Physicians and Surgeons of Ontario (CPSO) as of April 2014 [14]. Ontario is Canada’s largest province and accounts for more than one-third of the country’s 35 million population and approximately half of its over 500 dermatologists [15]. Dermatologists were excluded if they were no longer practicing dermatology in Ontario or if no contact information for their practices was available. Dermatologists with the same primary practice location were grouped together. A scripted telephone call was placed from an individual posing as a staff member from a referring physicians office or as a patient (for cosmetic services) to these primary practice locations over a two-month period, from May 6 –July 2, 2014, on at least three separate occasions. On each occasion, the receptionist answering the phone was asked for either: (1) the next earliest appointment for a routine skin exam–which was a service offered by all dermatology offices; (2) an urgent appointment for a changing mole suspicious for melanoma; or (3) the next earliest appointment for botulinum toxin injection for forehead wrinkles. These questions were asked as a proxy for Non-urgent, Urgent, and Cosmetic appointment times, respectively. Where date ranges were given, the mid-point was determined. All appointments offered were declined. Phone calls for Urgent care appointments were repeated for a randomly selected 5% of the of the population-wide sample, 6 months after the original telephone calls were made to further validate our data collection.

**Fig 1:**
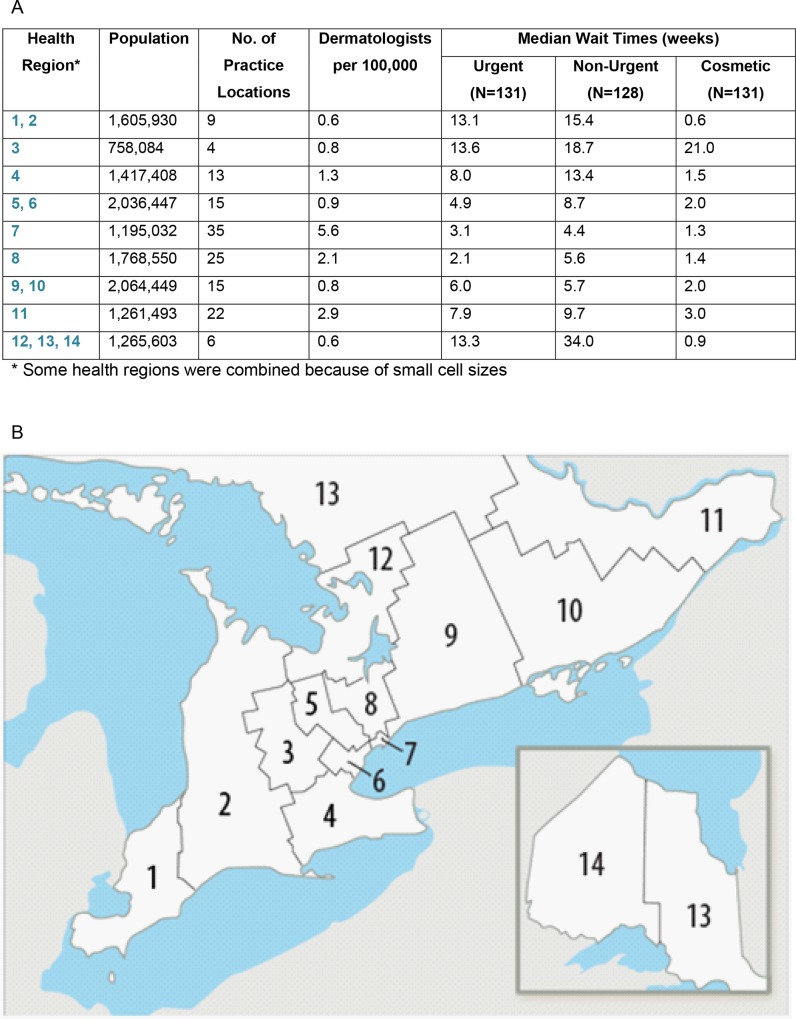
(A) Median Wait times by physician demographics and regional health authority* [16] (B) Map of regional health authorities produced by Ontario Local Health Integration Networks [17].

**Fig 2:**
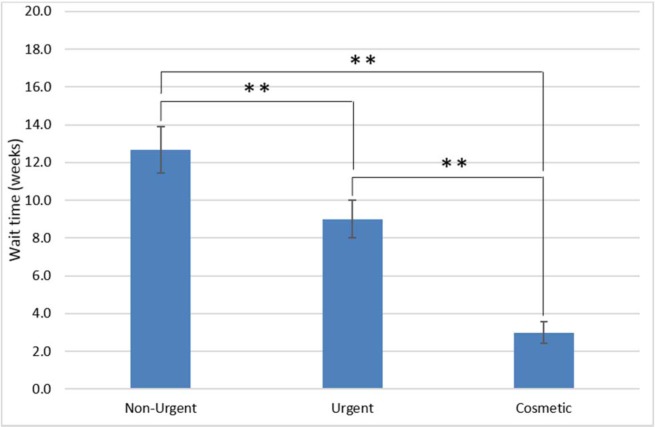
Wait times for dermatologic care across the province of Ontario for access to Non-Urgent, Urgent, and Cosmetic dermatologic services. Errors bars reflect Standard Error of the Mean (sem). **P <0.001.

**Fig 3:**
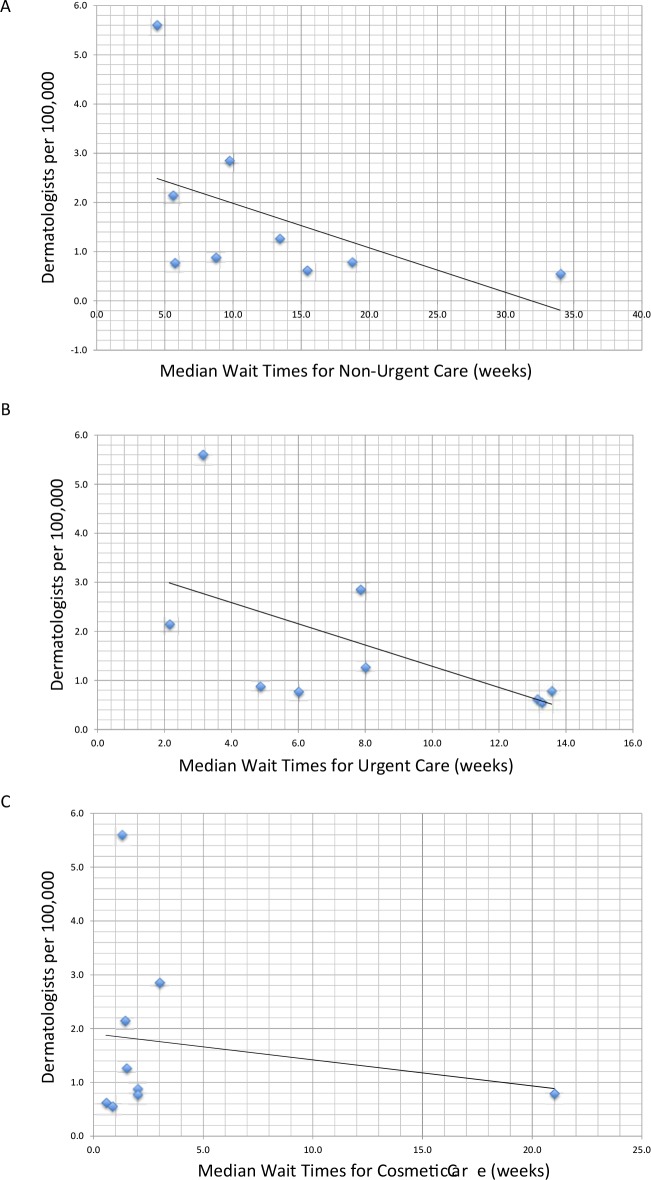
Median wait times for (A) Non-Urgent, (B) Urgent, and (C) Cosmetic care according to the density of dermatologists in each of nine health regions.

**Fig 4:**
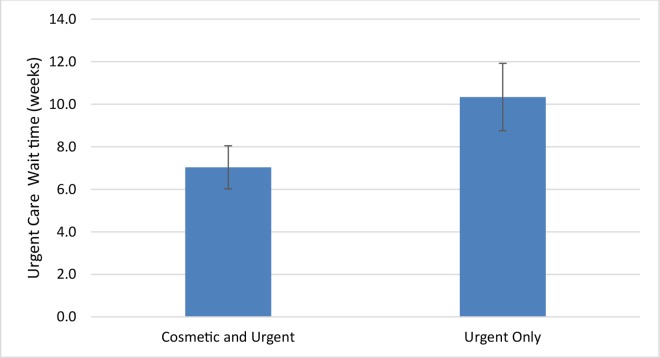
Urgent Care wait times for dermatology practices offering Cosmetic and Urgent care vs. those offering only Urgent care. Errors bars reflect Standard Error of the Mean (sem).

Descriptive statistics were used to express differences between groups. We used published regional data to calculate the density of dermatologists per 100,000 population by health region (Fig 1A) [16]. A one-way between-group ANOVA and Bonferroni-corrected post-hoc comparisons were conducted to test for differences in wait times among the three types of care: Non-Urgent, Urgent, and Cosmetic (Fig 2). The relationship between density of dermatologists and median wait time within each region (as indicated by a row in Fig 1A) for the different types of care was assessed using Spearman rank-correlation (Fig 3). In regions where the number of dermatologists was less than four, we combined that health region with the next nearest one geographically (Fig 1B) [17]. Finally, an independent samples t-test was conducted to compare wait times to receive Urgent care between those practices offering only Urgent care (i.e. those who did not offer botulinum toxin injections for forehead wrinkles) to those offering both Urgent and Cosmetic care (Fig 4). Wait times were measured in weeks from the date of the call until the date of the appointment offered. All data were analyzed using SPSS v22.0 for Windows (SPSS Inc., Chicago, IL, U.S.A). The Research Ethics Board of Mount Sinai Hospital approved the study protocols including the use of deception to waive written and verbal informed consent from participants. Declaring this as a research study may have led to bias and offering of an earlier appointment than normal.

## Results

From the 265 registered dermatologists in Ontario, 216 met the eligibility criteria and were classified into 144 primary practice locations, coded by regional health authority (Fig 1). Response rates were 89% for Non-Urgent consultations (n = 128), 91% for Urgent consultations (n = 131), and 91% for Cosmetic care (n = 131). Just over half (69/131; 53%) of the offices contacted for Cosmetic services had a dermatologist providing botulinum toxin injections. Of the primary practice locations we contacted 7 were no longer accepting new referrals and 3 provided only cosmetic dermatology services.

A one-way between-subject ANOVA was conducted to test for differences in median wait time among the three types of care (Urgent, Non-Urgent, Cosmetic). We found that wait times significantly differed across the three types of care (F(2,292) = 17.5, P < 0.001) (Fig 2). Bonferroni-corrected post-hoc comparisons indicated that the wait times across the three types of care were all significantly different from each other (all P < 0.05). Cosmetic care was associated with the shortest wait times (3.0 weeks; SD = 4.8; Interquartile Range (IQR) = 0.4–3.4), followed next by Urgent care (9.0; SD = 10.8, IQR = 2.1–12.9), and then by Non-Urgent care (12.7; SD = 12.8; IQR = 4.4–16.4). Additionally, only 24% of practices had an Urgent wait time < 2.0 weeks and 27% of practices actually had an Urgent wait time > 12.0 weeks. By contrast, 60% of practices had a Cosmetic wait time < 2.0 weeks. We re-assessed wait times for Urgent care in 5% of the eligible practices, 6 months after the original sample was completed. Median and mean wait times were similar across these time points.

We next investigated the relationship between the density of dermatologists and the median wait times in each of the regions (i.e. the rows depicted in Fig 1A) across care types using Spearman rank-correlation coefficients (Fig 3). This revealed significantly lower median wait time for Non-Urgent (r_s_ = -0.73, P < 0.05) and Urgent care (r_s_ = -0.69, P < 0.05) in areas of higher dermatologist density. We did not observe significant differences in wait times for Cosmetic care and dermatologist density (r_s_ = 0.26, P = 0.50), a relationship which still held when the one outlier was removed (Fig 3C).

Urgent wait times for practices that did not offer Cosmetic care (n = 62) were not different from practices offering both Urgent and Cosmetic care (N = 69) (10.3 vs 7.0 weeks; P = 0.07) (Fig 4).

## Interpretation

Our study examined the wait times for Urgent and Non-Urgent medical dermatologic care and compared them to wait times for Cosmetic dermatology services. Overall we found longer wait times and greater variation for medical dermatologic care and shorter wait times and less variation for cosmetic care. Not a single health region and only 24% of individual practices met the benchmark wait time of less than 2.0 weeks for Urgent care (our proxy for a suspected highly aggressive malignant disease) and 27% of practices had an Urgent wait time > 12.0 weeks [18, 19]. Overall, wait times for medically urgent dermatologic consultations were too long, especially when compared to cosmetic services.

Unlike previous surveys, we used a contemporaneous, population-wide approach of simulated calls to dermatology practices to assess whether wait times for medical care were affected by the offering of cosmetic services. Previous studies have reported much shorter wait times for cosmetic dermatologic care when compared to medical care [11, 12]. Though this was consistent with our own findings, the co-existence of medical and cosmetic services in the same practice was not associated with longer wait times for Urgent referrals. Two possible explanations are that these practices are more often situated in urban areas and that dermatologists offering cosmetic services have access to more resources that could create efficiencies in how quickly patients are seen (e.g. nursing staff). This finding, when considered alongside the significantly shorter wait times for Cosmetic care, suggests that though there may be some extra capacity in urban centers to see medical dermatology patients, the provision of cosmetic services does not negatively impact access to urgent dermatologic care.

Medically underserviced populations such as those living in rural areas can have especially poor access to specialist care and face long wait times to access that care [20, 21, 22]. Unsurprisingly, we found that wait times for Urgent and Non-Urgent care are significantly longer in those health regions with the lowest population density and the lowest dermatologist densities. In health regions where the dermatologist density was less than 1.0 per 100,000, Urgent median wait times were over 4.9 weeks and as high as 13.6 weeks (Fig 1A).

A shortage of dermatologists and increasing demand for their services raises concerns about dermatology workforce adequacy [23, 24]. Recent physician surveys have reported that access to dermatologic care in North America is limited. A 2007 survey of dermatologists collected by the American Academy of Dermatology found that although wait times for new patient appointments were slightly shorter when compared to 2002, the wait times in rural locations and for those practicing primarily medical dermatology were significantly longer [22]. In 2010, the Canadian National Physicians Survey reported that 37% of dermatologists in Ontario felt their accessibility to patients was either “poor” or “fair” and self-reported wait times for a non-urgent and urgent referral was reported as 12 weeks compared to 2 days, respectively [25].

Our study is among the first population-wide sample of wait times for dermatologists with near complete response rates. It is a contemporaneous examination of the impact of cosmetic services on timely access to care that was designed to reduce the effect of social desirability bias inherent in previous self-reported physician surveys. It employed a rigorous methodology that has been used in many previous studies regarding access to care [26, 27, 28, 29, 30, 31]. Still, our work has limitations. We were unable to ensure wait times were for the same practitioner in practices with multiple dermatologists when calling again for the other appointment types. However, our resample findings did demonstrate similar overall results and this methodology simulated the patient perspective of the primary care provider office booking an appointment. An additional caveat is that our use of botulinum toxin injections as a proxy for the provision of cosmetic services may have limited the number of practices that qualified under the category of providing Cosmetic care. Not using a more common service such as removal of a benign nevus may have underestimated the proportion of dermatologists providing cosmetic services. Resource limitations also prevented us from using different patient demographics to evaluate if wait times would vary by these sub-groups (e.g. pregnant, young, or immunosuppressed). Finally, we recognize that if a primary care provider calling for an appointment actually spoke to the practicing dermatologist, the wait time for Urgent care might have been shortened. This, however, requires willingness on the parts of both parties, which is not often possible given the inherent time constraints of the physicians involved. On balance, we feel that the limitations do not invalidate our primary findings about wait times for different types of dermatologic care.

Access to urgent and routine medical dermatologic care certainly has room for improvement, particularly in rural regions. The large variation in wait times we observed, even within the same health region, suggests that any referral system for medical care–especially in the case of suspected malignancy like melanoma—would benefit from a more streamlined approach. Dermatology in North America is a relatively small specialty and would be an excellent focus for developing such a system. Additionally, the use of teledermatology and closely supervised physician assistants could help meet the demand and shorten the wait times in areas with low-population density and high demand [32]. The use of these resources has been studied to some degree but their application and ability to deliver quality care requires further discussion and consensus [33]. Finally, reexamining the relationship between cosmetic and medical dermatologic care in the future could help assess trends and inform a discussion on what additional policies are required to ensure access to medically necessary dermatologic care in the face of increasing need. In this way, we can improve the experience for dermatology patients so they can help get the right care, in the right place, at the right time.
